# The progress of organ protection mechanisms in sepsis

**DOI:** 10.3389/fimmu.2025.1729499

**Published:** 2026-01-20

**Authors:** Yuanjie Zhu, Junjie Cheng, Zheyang Sun, Lu Jiang, Min Li

**Affiliations:** Department of Intensive Care Unit, The Fourth Affiliated Hospital of School of Medicine, and International School of Medicine, International Institutes of Medicine, Zhejiang University, Yiwu, China

**Keywords:** disease tolerance, organ protection, sepsis, sepsis treatment, treatment

## Abstract

**Background:**

Sepsis is a devastating condition with a high mortality rate, having garnered significant attention from physicians and scientists. Defined as life-threatening organ dysfunction resulting from a dysregulated host response to infection, sepsis poses a serious global health threat. Current treatment strategies for sepsis often remain inadequate and ineffective, increasing the burden on healthcare workers. Moreover, early detection of sepsis remains challenging, as existing scoring systems are ineffective in identifying and assessing pre-hospital sepsis. Therefore, early recognition and screening of sepsis symptoms are crucial for reducing the mortality rate and improving clinical outcomes.

**Main body:**

Sepsis precipitates multi-organ dysfunction through mechanisms including dysregulated inflammation, immunosuppression, metabolic derangement, and endothelial injury. Multiple organ failure remains a leading cause of death in severe sepsis cases. Understanding how to improve the condition of sepsis is of great significance. Scientists and physicians have demonstrated that some organs possess intrinsic protective mechanisms against sepsis-induced damage, which is part of the disease tolerance mechanism.

**Conclusion:**

This review focuses on exploring organ protection mechanisms in sepsis, centered on four pivotal axes, inflammatory regulation, immune homeostasis, metabolic adaptation and endothelial integrity. While current research has begun to unravel these mechanisms, translating these findings into clinically effective therapies remains a promising challenge.

## 1 Background

Sepsis represents a leading global health problem, formally defined as infection-induced life-threatening organ dysfunction stemming from maladaptive host responses (1) (**Table 1**). Epidemiologically, this disease accounts for more than 50% of in-hospital deaths among adults, directly causing 66% of fatal outcomes in affected hospitalizations (3). The global burden of sepsis is staggering. In 2017 alone, more than 11 million people died from sepsis and the number of sepsis cases was twice what was previously estimated (4). And it only calculated the cases in the hospital, there are 31 million sepsis cases all over the world (5), excepting the humans who fail to come to the hospital. Besides, severe sepsis not only significantly increases the risk of death but also has a profound negative impact on the long-term quality of life of survivors (6). Sepsis is characterized by its ability to cause damage to multiple organs. It disrupts cellular energy metabolism and leads to organ dysfunction, affecting vital organs (7), like the brain, heart, liver, kidneys and so on (8). With the present data, it proves that the main cause of severe death is multiple organ failure (9). Understanding the mechanisms by which organs protect themselves against sepsis-induced damage is crucial for developing more effective treatment strategies. According to recent researches, there are two main reasons to cause the sepsis. First, some non-infectious diseases later turn to shock and multiorgan failure, such as tissue ischemia, surgical tissues injury, pancreatitis, burns, vasculitis and drug reaction. And some of them subsequently develop evidence of infection (10). Second, most of sepsis is caused by infectious diseases. According to the data conducted across 1265 ICUs in 75 countries during a single day in May 2007 (11), the lungs constituted the most frequent site of infection (64%), with subsequent occurrences observed in the abdomen (20%), bloodstream (15%), and renal/genitourinary tract (14%) (10). According to another study analyzed in 2020, it reported that drawing from 51 studies conducted across 22 nations within 4 WHO regions, the incidence rate of 189 hospital-managed adult sepsis cases per 100,000 person-years. The corresponding case fatality rate was calculated at 26.7% (12).

**Table 1:** Previous and recently revised definitions of sepsis and related syndromes.

1991 Consensus Conference (2)	
Diagnosis	Signs and symptoms
Systemic inflammatory response syndrome (SIRS)	Two of the following: Temperature >38°C or <36°C Heart rate >90 beats per minute Respiratory rate >20 breaths per minute or arterial CO_2_<32mmHg White blood cell count >12×10^9^l^-1^ or <4×10^9^l^-1^ or >10% immature forms
Sepsis	SIRS with infection (presumed or proven)
Severe sepsis	Sepsis and acute organ dysfunction
Septic shock	Sepsis with persistent hypotension after fluid resuscitation

Despite advancements in sepsis treatment, managing severe cases remains challenging due to limitations in addressing multi-organ dysfunction. Current therapies like antibiotics and hemodynamic support focus on systemic infection and circulation but often fail to protect organs from inflammation-driven injury, reflecting a “blood-centric” paradigm that overlooks dynamic organ-immune interactions—such as how vascular support might harm endothelial barriers or disrupt immune balance. Organ protection, a strategy enhancing intrinsic defenses like antioxidant pathways or metabolic adaptation, offers promise by limiting tissue damage without direct pathogen targeting. By bridging the gap between systemic therapy and localized organ needs, this approach could mitigate dysfunction driven by uncontrolled inflammation and endothelial injury. Besides, Disease tolerance presents that organisms try to keep the stressor in a controllable range without influencing the pathogen load (13, 14) (**Table 2**). Sepsis-induced organ damage is well-documented, emerging evidence highlights intrinsic protective adaptations that may mitigate these effects. Recent research has shown that some organs have inherent protection mechanisms, which are part of the body’s disease tolerance strategy. This defense strategy can reduce the negative impact of infection (15). However, translating it to clinical use requires overcoming challenges in biomarker development, patient stratification, and designing interventions that balance infection control with tissue preservation—key steps to addressing current treatment gaps and improving sepsis outcomes.

**Table 2:** Difference between resistance and tolerance (15).

Conception	Resistance	Tolerance
Definition	Protects the host by reducing the pathogen burden.	Reduces the negative impact of infection on host fitness without affecting pathogen burden.
Primary Mechanism	Immune-mediated detection, neutralization, destruction, or expulsion of pathogens.	Reduces host susceptibility to tissue damage; involves stress responses, neural mechanisms, genetic programs.
Effect on Pathogen Burden	Directly reduces pathogen burden.	No direct effect on pathogen burden.
Impact on Host Fitness	Can reduce host fitness due to immunopathology (collateral tissue damage).	Preserves or improves host fitness by minimizing damage, enabling survival.
Costs Effects	Leads to immunopathology (e.g., tissue damage, compromised organ function); cost correlates with immune response magnitude/duration.	Mechanisms may involve resource allocation, but the main effect is damage reduction; allows for a stronger immune response without increased damage.
Role of the Immune System	Central role, the immune system directly clears pathogens.	Indirect or partial role (e.g., phagocytosis); extends beyond immunity to general physiological processes.
Evolutionary Trade-off	Constrained by a trade-off with immunopathology, optimal response balances clearance and acceptable damage.	De-constrains the immunity-immunopathology trade-off, allowing for a more robust immune response.

This review systematically summarizes the protective regulatory mechanisms of the hematopoietic system, cardiovascular, hepatic, pulmonary, renal, gastrointestinal, and neurological systems in sepsis, focusing on four key regulatory axes: suppressing excessive inflammatory responses, regulating and restoring immune homeostasis, improving cellular metabolic dysfunction, and enhancing endothelial barrier stability. Although existing studies have partially elucidated organ-specific protective mechanisms, a critical gap persists in systematic cross-organ comparative research. Further exploration of the shared and unique features of organ-specific protection mechanisms may reveal novel therapeutic targets for sepsis management. This systematic analysis not only advances our understanding of sepsis pathology but also bridges fundamental research with clinical applications, ultimately contributing to reduced mortality and improved prognostic strategies in sepsis care.

## 2 The mechanism of organ dysfunction caused by sepsis

**Figure 1:**
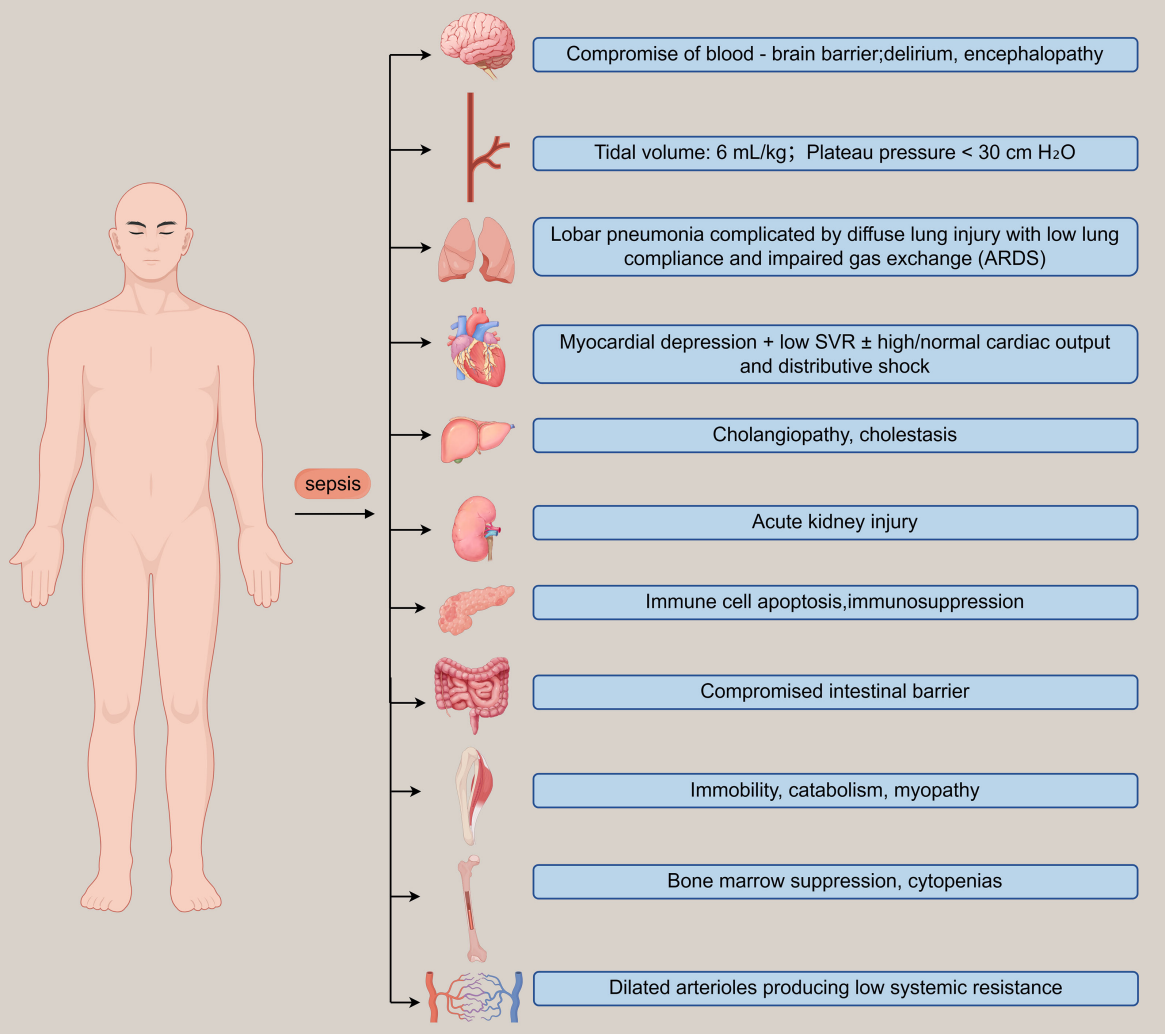
Organ failure in a critically ill patient with septic shock.

As previously discussed, sepsis is a systemic pathological process triggered by pathogen-induced infections. This life-threatening condition affects multiple organ systems through several interdependent pathways, including the dynamic imbalance between hyperinflammation and immunosuppression, metabolic dysregulation, and endothelial dysfunction. These interconnected mechanisms collectively contribute to the complex pathophysiology of sepsis (**Figure 1**).

### 2.1 Excessive inflammatory response

Under severe systemic infection, internal or external perturbations can cause key regulated variables to deviate significantly from their set points, overwhelming negative feedback mechanisms and thereby disrupting immune homeostasis (16). The resulting disruption of immune homeostasis defines the core of immunopathology (15). Sepsis is typically initiated by bacteria or other pathogens that trigger an uncontrolled inflammatory response in the body (17). Particularly in the early stages of sepsis, receptor activation induces a potent inflammatory reaction capable of causing host damage (18). Hyperinflammation arises from the dysregulated activation of pro-inflammatory effector mechanisms—such as activated leukocytes and endothelial cells—accompanied by aberrant production of oxygen/nitrogen radicals and cytokines, along with dysregulation of the complement system and coagulation cascade. Although these mechanisms fundamentally represent the body’s innate immune defense against infection—mediated by a homeostatic balance between inflammatory and protective responses (19). Severe sepsis is associated with an even more intense inflammatory response, characterized by elevated cytokine levels and progressive organ dysfunction (20). Neutrophils and neutrophil extracellular traps (NETs) activate the NF-κB pathway in endothelial cells, triggering pro-inflammatory and pro-angiogenic responses that further disrupt immune regulation (21). This activation enhances glycolysis in endothelial cells, driving pro-inflammatory programs and leading to the accumulation of reactive oxygen species (ROS). Consequently, a vicious cycle is formed that perpetuates vascular inflammation and exacerbates oxidative stress (22). Using a murine sepsis model, Wang et al. demonstrated that NETs promote sepsis-induced acute lung injury (SI-ALI) by augmenting ferroptosis in alveolar epithelial cells (23). Furthermore, endothelial cell-derived extracellular vesicles (EC-EVs) are selectively internalized by pulmonary monocytes, driving their transdifferentiation into pro-inflammatory macrophage subtypes. This cellular reprogramming precipitates a cytokine storm and amplifies inflammatory cell infiltration, thereby aggravating pulmonary tissue damage (24). During sepsis, the complement is activated. Complement activation leads to the release of small fragments called anaphylatoxins, notably C3a and C5a. These molecules exert potent pro-inflammatory effects by recruiting and activating leukocytes, endothelial cells, and platelets (25). While complement activation is an essential component of protective immunity, uncontrolled activation can be highly damaging, resulting in tissue damage and organ failure.

### 2.2 Immunosuppression

Sepsis also induces a state of immunosuppression, which significantly increases susceptibility to secondary infections and contributes to high mortality rates. This immunosuppressive phase is characterized by a marked decline in lymphocytes and diminished expression of human leukocyte antigen DR (HLA-DR), limiting the efficacy of immune-stimulating therapies (26). A central feature of sepsis-induced immunosuppression is the depletion of key immune cells, including CD4+ T cells and B cells (27, 28). This lymphopenia is largely driven by extensive apoptosis of immune cells (29). Additionally, the expansion of myeloid-derived suppressor cells (MDSCs) further suppresses T-cell function and promotes an immunosuppressive microenvironment (30–32). Several molecular mechanisms underpin this immunoparalysis. The cholinergic anti-inflammatory pathway, mediated by the vagus nerve and acetylcholine, attenuates pro-inflammatory cytokine release but may contribute to immunosuppression in later stages (33). The studying, it proved that high-mobility group protein B1 (HMGB1) performed a function in immunosuppression in the late phase of sepsis and defective neutrophil, which increases the secondary infections (34). Current studies show that programmed cell death receptor-1 (PD-1) exerts an influence on immunosuppressive progress (35). The simultaneous release of both pro- and anti-inflammatory mediators leads to adaptive immune suppression through feedback inhibition. This is manifested as reduced HLA-DR expression, enhanced PD-1/PD-L1 signaling, accelerated immune cell apoptosis, and T-cell exhaustion (36). There is another theory that endotoxin tolerance leads to immunosuppression. When the immune cells are exposed to a low dose of pathogen, they fail to resist the endotoxin (37). For instance, dendritic cells shift toward a tolerogenic phenotype after infections such as pneumonia, increasing the risk of secondary complications (38). The immunodynamic progression of sepsis often follows a biphasic pattern: an initial hyperinflammatory state is followed by a dominant immunosuppressive phase, the latter being characterized by defective antimicrobial defenses (39, 40). Neutrophils exemplify this dysfunction—during severe sepsis, they often exhibit impaired migration to infection sites (22, 41), and their upregulation may paradoxically contribute to immunosuppression (42).

### 2.3 Metabolism disorder

The execution of specialized immune functions during infection not only requires energy but also depends critically on anabolic metabolism to support large-scale biosynthetic demands. Sepsis profoundly disrupts cellular metabolism. It disrupts lipid metabolism, leading to a decrease in lipid levels, and also causes energy metabolism disorders (43). Early in sepsis, metabolic alterations such as changes in amino acid levels can occur even before significant liver dysfunction is apparent. These amino acid imbalances are not merely epiphenomena but may actively contribute to organ dysfunction, including the development of septic encephalopathy (44). Numerous studies have shown that sepsis is associated with hypocholesterolemia, and the severity of this condition is a strong predictor of poor outcomes. Sepsis affects cholesterol synthesis, transport, and metabolism, which in turn affects its biological functions, including immune function, hormone and vitamin production, and cell membrane receptor sensitivity (45). Concurrently, the inflammatory functions of macrophages rely on aerobic glycolysis and anabolic programs. Specifically, Lipopolysaccharide (LPS) stimulation activates mammalian target of rapamycin (mTOR) in macrophages, upregulates μPFK2, and thereby promotes aerobic glycolysis. In a coordinated manner, LPS triggers the mTOR–HIF1α pathways, which elevate GLUT1 transcription to support aerobic glycolysis (46). Far from being a mere metabolic waste product, lactate exacerbates sepsis-induced vascular hyperpermeability by activating the GPR81 receptor on endothelial cells and disrupting tight junction integrity (47). These metabolic shifts reflect a fundamental physiological conflict during systemic infection. The host mounts a vigorous immune response (resistance) that is inherently anabolic, consuming energy to synthesize effector molecules and cells. This organism-wide anabolic state, however, may come at the cost of disease tolerance. Tolerance mechanisms, which are catabolic and depend on energy from macromolecule breakdown to preserve tissue structure and function, can be substantially impaired by this competition for energetic resources (46). Consequently, the organism’s ability to maintain homeostasis hinges on precisely regulating the dynamic interplay between anabolic (hypermetabolic) and catabolic (hypometabolic) programs in response to internal signals, a mechanism that is fundamentally dysregulated in sepsis (48).

### 2.4 Endothelial dysfunction

**Figure 2:**
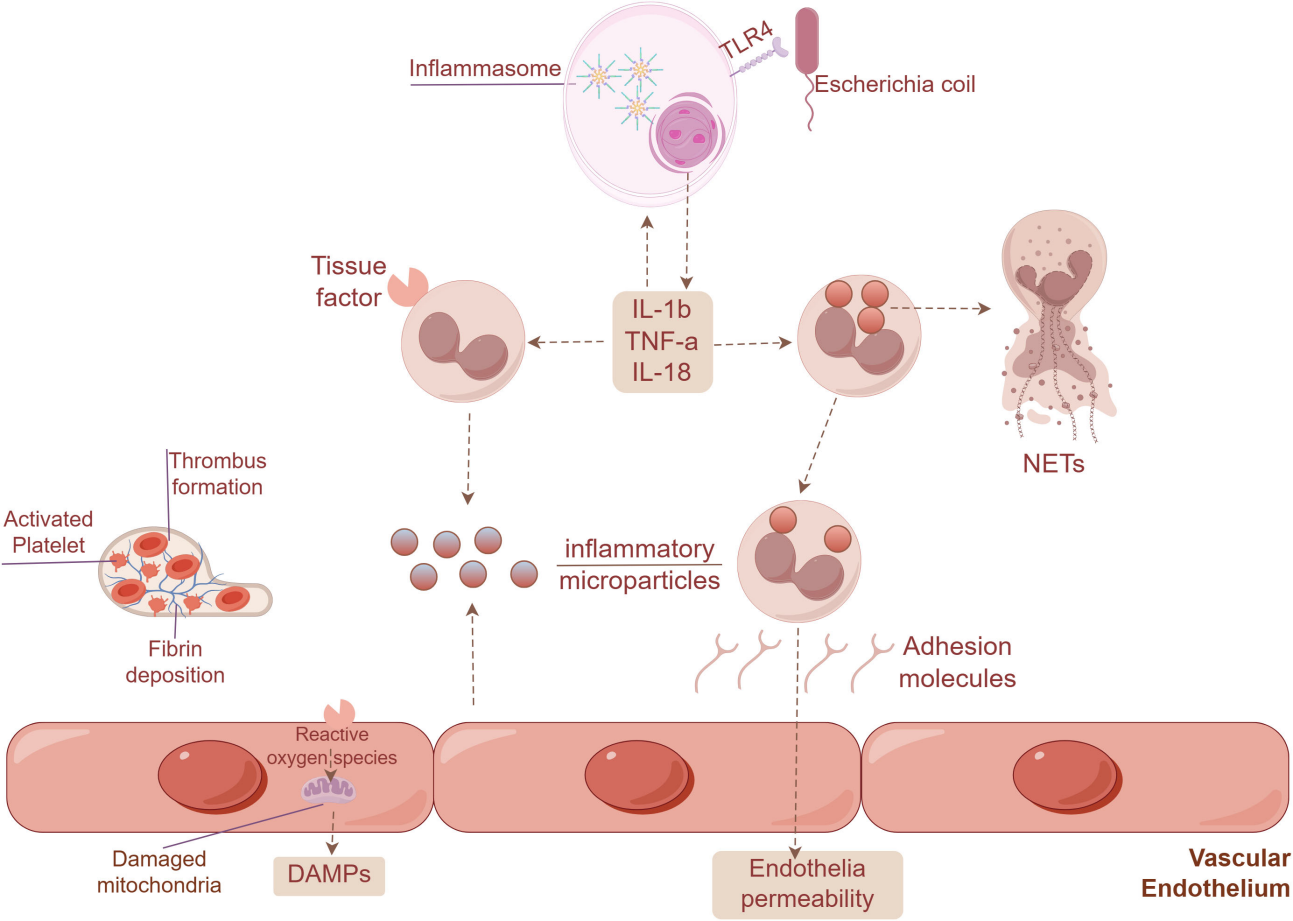
Endothelial injury in sepsis drives a self-reinforcing pathophysiologic cycle, characterized by monocyte and granulocyte activation, endothelial barrier breakdown, immunothrombosis, and disseminated intravascular coagulation.

Endothelial dysfunction is a central driver of sepsis pathology, directly contributing to critical conditions such as vascular leakage, refractory hypotension, and multiple organ dysfunction (49). This dysfunction manifests through a spectrum of pathophysiological features, including degradation of the endothelial glycocalyx (eGC), disruption of intercellular adherent junctions, and excessive production of chemokines, which collectively promote capillary hyperpermeability and aberrant leukocyte recruitment (50). A key initiating mechanism is the sepsis-induced overproduction of angiopoietin-2 (Ang-2), which competitively inhibits the protective TIE2 signaling pathway in endothelial cells. This Ang-2/TIE2 axis disruption triggers a pathologic metabolic shift, characterized by the overactivation of aerobic glycolysis. This shift directly impairs vascular barrier function and alters the expression of adhesion molecules, ultimately disrupting the coordinated recruitment of immune cells to sites of infection (51). The metabolic reprogramming of endothelial cells extends beyond glycolysis. And through a proteomics-based metabolic model, it was observed that upon complete formation of the vascular network, fatty acid oxidation (FAO) in endothelial cells is upregulated, whereas glycolysis is downregulated. Inhibition of carnitine palmitoyltransferase 1A (CPT1A), the enzyme responsible for facilitating the transport of fatty acids into mitochondria for oxidation, leads to increased endothelial cell permeability (52). These injurious processes are amplified by a cascade of inflammatory mediators. Systemic inflammation rapidly induces disintegration of the structurally fragile eGC. This degradation unmasks procoagulant domains on the endothelial surface and triggers the overexpression of tissue factor (TF) and von Willebrand factor (VWF), collectively promoting a pro-coagulant state (53). The ensuing leukocyte-platelet adhesion and reduced capillary flow velocity predispose to microthrombi formation and capillary blockage. In organs such as the kidney, this cascade leads to ischemic and inflammatory damage, contributing to acute kidney injury (54). Furthermore, elevated levels of IL-22BP during sepsis further amplify inflammation and impair both endothelial and epithelial barriers, creating a vicious cycle of injury and dysfunction (55). Collectively, these cascading events form a self-reinforcing cycle of endothelial injury that is central to sepsis pathophysiology, characterized by sustained leukocyte activation, loss of barrier integrity, immunothrombosis, and progression toward disseminated intravascular coagulation (**Figure 2**).

Sepsis is a systemic dysregulation triggered by infection, which is primarily a disorder of the host’s own response. This is evidenced by the fact that many patients succumb even after antibiotics have eradicated the pathogen (56). Throughout its progression, the condition exhibits a dynamic biphasic imbalance. The early phase is dominated by hyperinflammation. As the condition progresses, the host shifts toward profound immunosuppression, creating an “immune paralysis” window prone to secondary infections. Simultaneously, systemic metabolic collapse persists across all stages, these metabolic disturbances not only exacerbate organ injury but also serve as critical prognostic biomarkers. Notably, progressive endothelial barrier dysfunction and vascular leak syndrome directly compromise microcirculatory homeostasis, leading to multi-organ hypoperfusion. Having a basic understanding of the pathophysiology of sepsis, next, we will introduce how the organs protect themselves during sepsis.

## 3 Organ protection mechanism

Current research has identified core protective mechanisms in organ preservation, primarily encompassing four key aspects: modulation of inflammatory responses, restoration of immune homeostasis, enhancement of cellular metabolism, and maintenance of endothelial barrier integrity. These mechanisms collectively address critical pathological processes observed in sepsis progression.

### 3.1 Inflammatory response regulation

Organs have evolved various strategies to counteract the damage caused by sepsis. Inflammation, while a contributor to pathology, is fundamentally a protective program that mobilizes emergency responses to restore homeostasis amid severe physiological disruption (57). Initially, they trigger an inflammatory response to eliminate the pathogen. The blood system mobilizes immune cells. These cells play their unique roles in confronting the pathogen.

#### 3.1.1 Adaptive immunity and lymphocyte role

The adaptive immune system, particularly lymphocytes, plays a previously underappreciated role in limiting tissue injury. Research building on the long-term protective effects of LPS pre-exposure revealed that in tolerant hosts, lymphocytes are central to mediating tissue protection, independent of bacterial load. This was demonstrated in Rag2^−^/^−^ mice, where lymphocyte deficiency abolished LPS-induced protection—a defect that was reversed by adoptive splenocyte transfer (58). The clinical relevance is underscored by findings that Rag1^−^/^−^ mice, which lack B and T cells, exhibit higher mortality after cecal ligation and puncture (CLP), and that B cells contribute to early protective innate responses during bacterial sepsis (59).

#### 3.1.2 Innate immune mechanisms

Innate immune cells also contribute to protection through multiple mechanisms. Neutrophils combat pathogens via phagocytosis, degranulation, and the release of neutrophil extracellular traps (NETs), which can trap circulating bacteria and limit dissemination in models of E. coli sepsis (60, 61). Beyond their antimicrobial functions, NETs can also modulate adaptive immunity: they drive metabolic reprogramming in naïve CD4^+^ T cells through the Akt/mTOR/SREBP2 signaling pathway, resulting in enhanced cholesterol biosynthesis. This metabolic shift promotes the differentiation of CD4^+^ T cells into regulatory T cells (Tregs) and augments their suppressive function (62). NETs also anchor enolase 1 (ENO1) to the membrane of CD4^+^ T cells via their key component myeloperoxidase (MPO), subsequently recruiting interferon-induced transmembrane protein 2 (IFITM2). IFITM2 functions as a DNA sensor that recognizes NET-derived DNA, triggering activation of intracellular RAS-associated protein 1B (RAP1B) and its downstream Extracellular signal-regulated kinases (ERK) signaling pathway, thereby promoting the differentiation and functional capacity of Tregs (63). Moreover, certain innate immune cells and stromal populations actively regulate inflammation and support tissue repair. For example, adipose-derived mesenchymal stromal cells (AD-MSCs) improve survival and attenuate organ injury in experimental sepsis by secreting interleukin-10 (IL-10), which dampens pro-inflammatory cytokine production in macrophages (64).

#### 3.1.3 Molecular regulators

At the molecular level, precise regulatory programs guide the immune response. The zinc finger protein tristetraprolin (TTP) exerts stage-specific functions: early in inflammation, it suppresses pro-inflammatory cytokines by binding AU-rich elements in mRNA 3’-UTRs; later, Sirt1-mediated deacetylation enhances its capacity to promote bacterial clearance via autophagolysosome formation, revealing a metabolic-epigenetic axis in inflammation resolution (65). Moura-Alves et al. found that aryl hydrocarbon receptor (AhR) can detect various bacterial virulence factors and regulate antibacterial responses. AhR demonstrates dual functionality in antimicrobial defense, coordinating both virulence factor neutralization and bactericidal clearance. Immune cells employ pattern recognition receptors (PRRs) like Toll-like receptors (TLRs) to detect pathogen-associated molecular patterns (PAMPs), triggering host defense mechanisms against microbial invasion. It identified bacterial pigments—specifically phenazines and naphthoquinones—as a novel class of PAMPs and redefine AhR as a pattern recognition receptor. This expanded definition positions AhR as a multimodal sensor capable of detecting both microbial-derived PAMPs and endogenous danger-associated molecular patterns (DAMPs) generated during inflammatory stress. Such dual sensing capability establishes AhR as a critical immune surveillance mechanism for orchestrating antibacterial responses, bridging pathogen recognition and host-derived danger signal integration to control bacterial infections (66).

Systemic and vascular mechanisms further contribute to host resilience. Endothelial microparticles (EMPs) are beneficial proinflammatory substances that can improve the survival rate of septic patients. Comparative analyses demonstrated elevated concentrations of both unbound EMPs and EMP-monocyte conjugates in survivors relative to nonsurvivors. Increased monocyte-associated E-selectin (CD62E) levels emerged as an early and sustained biomarker predictive of reduced mortality risk and attenuated organ dysfunction. This inducible endothelial adhesion molecule, expressed on activated vascular endothelium, may associate with monocytes through two potential mechanisms: direct cellular interactions with vascular endothelium facilitating membrane protein transfer, or enhanced adsorption of soluble CD62E+ EMPs by activated monocytes. Alternatively, differential microvascular sequestration of these complexes in nonsurvivors may account for the observed disparity (67). Pan et al. demonstrated that stimulating the vagus nerve can reduce the expression of pro-inflammatory cytokines and improve symptoms such as hypotension and disseminated intravascular coagulation caused by sepsis (68). Also, neuroimmunological pathways also offer therapeutic insights. The sphingolipid sphingosine - 1 - phosphate signaling pathway plays a significant role in maintaining the integrity of the central nervous system and mediating neuroinflammation (69).

### 3.2 Restoration of immune homeostasis

During the progression of sepsis, the body employs multiple mechanisms to re-establish immune homeostasis and improve survival rates. Homeostasis is the stable internal state maintained by an organism through negative feedback mechanisms, which counteract fluctuations in the external environment and changes in internal activity (70).

#### 3.2.1 Molecular immune homeostasis

At the molecular level, persistent immune stimulation can induce alterations in histone modifications and affect chromatin remodeling machinery, ultimately leading to the LPS tolerance phenotype. Regulatory elements such as miRNAs further stabilize these changes by inhibiting key regulators in this process (71). Inflammation-related genes (e.g., TLR-4) also undergo reprogramming (72), while interferon-gamma (IFN-γ) can reverse the immunosuppressed state by enhancing the antimicrobial functions of mononuclear phagocytes, promoting pathogen clearance, and upregulating MHC I/II molecule expression to restore immune balance (73). p53 plays a critical protective role in host defense: it limits excessive production of pro-inflammatory cytokines (e.g., TNF, IL-6) by suppressing the NF-κB signaling pathway, thereby preventing hyperinflammation. It restricts macrophage overactivation by promoting their apoptosis, and it modulates neutrophil functions by inhibiting phagocytosis, oxidative bursts (via Nox2-dependent ROS), and protease release to mitigate tissue damage. Also, p53 balances pathogen clearance and tissue protection—enhancing bacterial elimination while dampening inflammatory responses to prevent pulmonary microvascular injury and immunopathology. By coordinating macrophage fate, neutrophil activity, and NF-κB signaling, p53 maintains a delicate balance between antimicrobial defense and tissue protection (74). Research revealed elevated Hypoxia-inducible factor 1-alpha (HIF-1α) activity and expression levels in sepsis-derived monocytes. Their findings suggest endotoxin exposure during sepsis initiates HIF-1α activation in monocytes, subsequently promoting IRAKM production and driving these immune cells toward an endotoxin-tolerant status. Furthermore, HIF-1α demonstrated dual regulatory effects - while enhancing IRAKM expression in monocytes, it simultaneously suppressed LPA-induced pro-inflammatory cytokine production, particularly TNF and IL-6. This will be beneficial to improve immune homeostasis (75).

#### 3.2.2 CNS-mediated immune homeostasis

The central nervous system (CNS) regulates innate immune responses through hormonal and neuronal pathways. The neuroendocrine stress response, along with the sympathetic and parasympathetic nervous systems, generally inhibits innate immunity systemically and regionally. Conversely, the peripheral nervous system often amplifies local innate immune responses. These systems work in tandem. First, the nervous system also influences the inflammatory response through hormonal and neuronal mechanisms. The engagement of innate immune defenses following pathogen detection initiates dual signaling pathways: pro-inflammatory cascade activation and concurrent neuroregulatory circuit stimulation to modulate inflammatory resolution. Peripheral neural networks at inflammation loci potentiate innate immunity through neuroimmune crosstalk, inducing vasoactive compound secretion that augments leukocyte infiltration and pathogen elimination efficacy. Microbial eradication triggers coordinated neuroendocrine responses mediated by hypothalamic-pituitary-adrenal axis activation coupled with sympathetic-parasympathetic modulation, collectively suppressing excessive inflammation and reestablishing physiological equilibrium (76). And Chavan reported that adrenergic neural circuits can regulate the protective program. Luminal bacterial infection activates extrinsic adrenergic neurons innervating the gut, triggering norepinephrine (NE) release. This adrenergic signal induces a tissue-protective phenotype in muscularis macrophages through β2-adrenergic receptor (ADRB2) signaling, thereby enhancing their capacity to mitigate infection-driven intestinal damage (77).

#### 3.2.3 Lung immune homeostasis

The lung has its unique immune-regulating mechanisms. It was proved that there was a binding called connexin 43 between alveolar macrophages and epithelial cells to transport the signals to avoid excessive immune response (78). The combined effects of AHR and IDO1 are necessary for LPS-triggered tolerance to downregulate early inflammatory gene expression (79). Sepsis can cause acute lung injury. In specific situations, autophagy may play a protective role in the onset and advancement of ALI. In the murine model of sepsis induced by CLP, decreased expression of LC3-II, ATG5, and ATG7 has been observed in pulmonary tissues, indicating potential suppression of autophagy during septic progression. Pharmacological enhancement of autophagy through rapamycin or activated protein C (APC) administration demonstrates anti-inflammatory effects and ameliorates pulmonary damage in this experimental setting (80). Pulmonary B1a cells prevent neutrophil infiltration and reduce MPO production in the lungs, protecting the organ (81). mesenchymal stem cells contribute to reducing all pulmonary cytokines and chemokines in the lungs during sepsis, which diminishes the lung injury. The mice in sepsis were coordinately modulated by MSC administration, exhibiting broad suppression of inflammatory genes alongside concomitant upregulation of genes supporting antigen presentation efficiency, phagocytic activity, and pathogen elimination. This equilibrium in inflammatory reprogramming during sepsis necessitates synchronized adjustments to pivotal innate immune pathways governing microbial defense mechanisms at critical regulatory nodes to modify the immune homeostasis (82).

#### 3.2.4 Cardiac immune homeostasis

In cardiomyocytes, sepsis-induced dysregulation of systemic inflammatory mediators triggers the maladaptive remodeling characteristic of sepsis-induced myocardial injury (83). Mesenchymal stem cells (MSCs) play a role in decreasing cardiomyocyte apoptosis during sepsis. Experimental evidence indicates that miR-223 plays a critical role in mediating the therapeutic benefits of MSCs on survival outcomes in polymicrobial sepsis. Mice with miR-223 exhibited to significantly attenuate cardiomyocyte apoptosis in CLP-induced septic mice, achieving a quantifiable reduction to approximately 3 apoptotic nuclei per 1000 in myocardial tissue compared to sham-operated controls (which showed 8 apoptotic nuclei per 1000) (84).

#### 3.2.5 Hepatic immune homeostasis

In the context of injury-induced immunosuppression, immature hepatic natural killer T (NKT) cells, in conjunction with noradrenergic innervation, play a central regulatory role. This means that these cells, present in the liver in an immature state, are involved in a regulatory process that impacts the body’s immune response following injury. The regulation occurs centrally and is intertwined with the function of noradrenergic innervation, which likely modulates the overall immunosuppressive effect (85). Moreover, during the progression of sepsis, the functionality of cluster of differentiation 39 (CD39) is simultaneously crucial for the generation of adenosine, which has anti-inflammatory, immunosuppressive, and protective effects in sepsis-associated liver injury (86). Li et al. put forward that fibroblast growth factor 9 (FGF9) remarkably reduces the hepatic injury markers such as T-BIL, ALT, and AST, which promotes the protection of the liver (87).

### 3.3 Improvement of cellular metabolism

**Figure 3:**
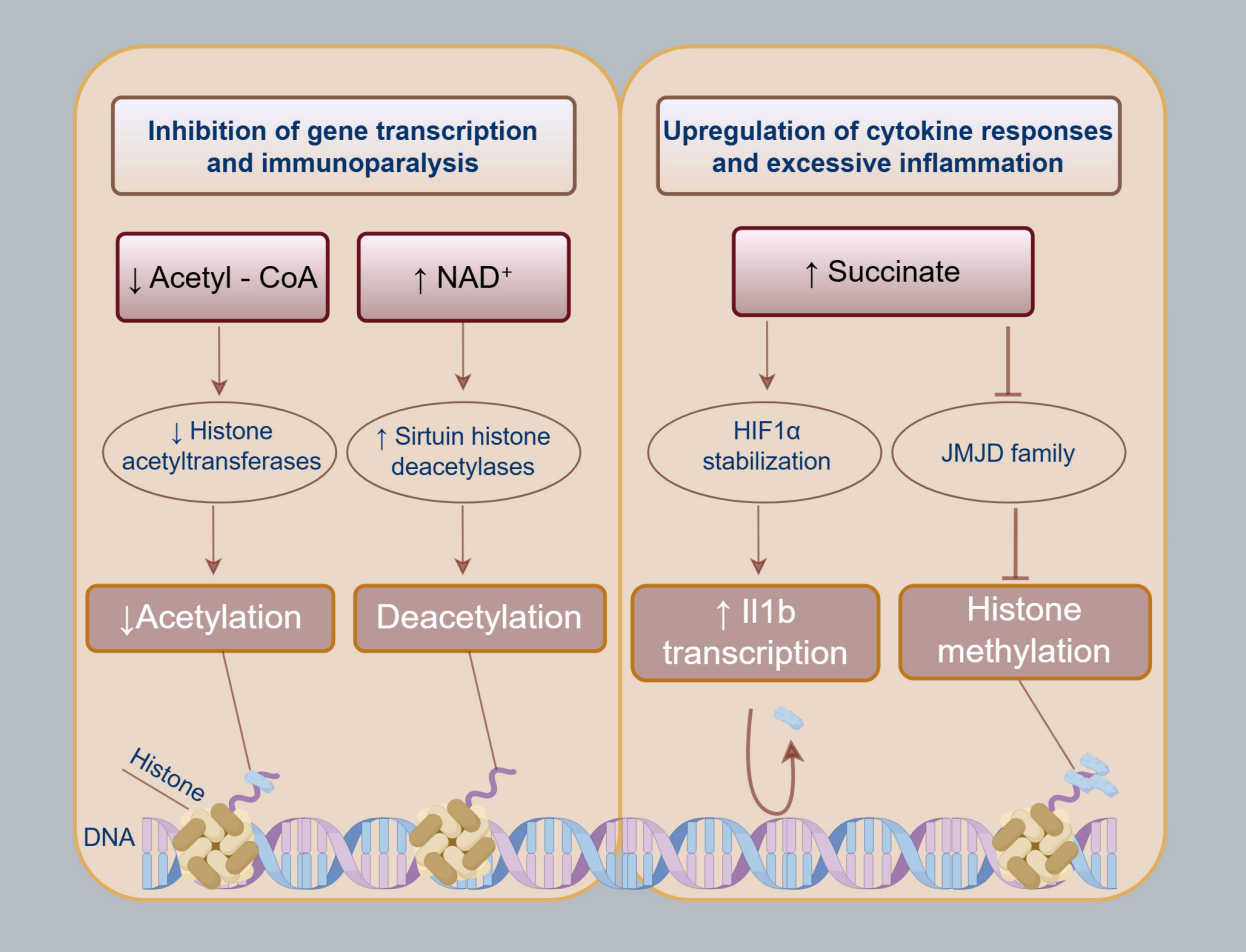
Metabolic and epigenetic pathways induce an imbalance in immune responses during sepsis. During sepsis, innate immune cell activation triggers intracellular signalling and cellular metabolic reprogramming. In the early phase, activated pathways like oxidative phosphorylation and glycolysis accumulate metabolites that serve as cofactors or modulators for epigenetic enzymes. These interactions induce chromatin modifications, altering gene transcription. Critically, specific metabolite shifts can drive divergent immune outcomes: Decreased acetyl-CoA or elevated NAD+ (activating sirtuins) reduces histone acetylation, inhibiting transcription and promoting immunoparalysis. Conversely, succinate accumulation in some patients stabilizes HIF1α and inhibits JMJD demethylases, enhancing cytokine responses and excessive inflammation. Thus, sepsis-associated metabolites regulate epigenetic enzymes, ultimately programming immune cells towards either immunoparalysis or hyperinflammation.

During sepsis, metabolic dysregulation aggravates organ dysfunction while metabolic reprogramming simultaneously activates protective mechanisms. Emerging evidence establishes that sepsis-associated metabolic reprogramming is a adjusting driver of immune imbalance and multi-organ failure (88). At the gene regulatory level, epigenetic modulation of gene activity has emerged as a critical regulatory pathway in orchestrating myeloid cell function during sepsis (**Figure 3**). This epigenetic regulation is mediated by chromatin remodeling processes that dynamically reconfigures gene-associated regions into transcriptionally permissive euchromatin or repressive heterochromatin states. Transcription-competent euchromatin allows access to transcriptional machinery including RNA polymerases and regulatory proteins, while transcriptionally restricted heterochromatin forms an impenetrable barrier that suppresses gene expression. A variety of histone post-translational modifications, including acetylation, methylation, ubiquitylation, and phosphorylation, critically influence chromatin accessibility and transcriptional activity. Metabolic alterations and epigenetic reprogramming synergistically contribute to the maladaptive immune responses characteristic of sepsis (56).

#### 3.3.1 Cardiac metabolism

In the heart, multiple metabolic adaptations support survival. GDF15 functions as an endocrine mediator that orchestrates inflammatory injury tolerance through dual mechanisms: preservation of myocardial performance and thermoregulatory stability, achieved via precise modulation of triglyceride metabolic pathways (89). Similarly, cardiac peroxisome proliferator-activated receptor alpha (PPARα) expression contributes to survival during sepsis by enhancing cardiac performance and fatty acid oxidation. The observed survival reduction in PPARα−/− murine models appears mechanistically linked to cardiac bioenergetic failure, principally stemming from compromised fatty acid β-oxidation capacity – a pathogenic determinant of myocardial substrate insufficiency. This pathophysiological evidence positions dysregulated PPARα signaling as a critical modulator in sepsis-induced terminal organ pathology, with particular emphasis on its cardiocentric role through disrupted metabolic homeostasis (90). LPS administration triggers mitochondrial dysfunction, which in turn robustly activates cardiac mitophagy. Cardiac mitophagy is likely beneficial, as it probably aids in restoring normal mitochondrial and cardiac function following sepsis-induced impairments (91). Mitochondrial dysfunction may activate pathological signaling pathways, induce impaired contractility in myocardial cells, as well as trigger cellular apoptosis in cardiomyocytes. And melatonin exerts its therapeutic effects through reducing NADH oxidase content, enhancing NADPH-cytochrome C reductase expression, and restoring mitochondrial functionality in cardiomyocytes, concurrently suppressing pro-inflammatory mediators (IL-1α, IL-1β, IL-6, Mcp-1) to mitigate sepsis-induced myocardial dysfunction (92).

#### 3.3.2 Hepatic metabolism

In the liver, purinergic signaling plays a protective role. CD39 plays a role in eliminating extracellular adenosine triphosphate (ATP), which is beneficial in limiting inflammation and damage. The purinergic signaling cascade triggered during sepsis operates through sequential enzymatic control. PRRs-mediated detection of pathogen-associated molecular patterns (e.g., bacterial lipopolysaccharide) initiates ATP efflux through P2X7 receptor stimulation. This receptor’s activation amplifies ATP liberation predominantly via pannexin-1 channels, creating a feedforward loop for purinergic signaling propagation. CD39 executes nucleotide triphosphate dephosphorylation to adenosine monophosphate, thereby attenuating P2X7 overactivation and suppressing inflammatory cascades. Subsequently, CD73-mediated dephosphorylation converts adenosine monophosphate (AMP) to adenosine, serving to counteract inflammation while enhancing cytoprotective mechanisms. This coordinated enzymatic cascade demonstrates operational efficacy in mitigating hepatic damage and facilitating homeostatic recovery during systemic infection (86). And in aged mice, a higher total content of hepatic fatty acids and an altered ratio of C18 to C16 may improve the immune responses in the liver and increase endotoxin tolerance (93). Ferritin H chain (FTH) is conducive to enhancing disease tolerance during sepsis by maintaining the expression/activity of the liver G6Pase to boost disease tolerance. FTH is essential for sepsis disease tolerance in mice, requiring its expression in hepatocytes and likely macrophages to execute this protective function. This tolerance mechanism operates through FTH-mediated coordination of glucose metabolic reprogramming, specifically by controlling hepatic glucose-6-phosphatase (G6Pase) complex expression and enzymatic activity during polymicrobial infection (14). Hepatic mitochondrial dysfunction is universe. While macrophage TREM2 plays a protective role in shielding hepatocytes from mitochondrial dysfunction to ameliorate disease progression. Hepatic mitochondrial dysfunction emerges as a critical mediator and its sepsis-related complications, with macrophage TREM2 identified as a protective factor counteracting hepatocyte mitochondrial impairment. Mechanistically, TREM2 governs hepatic lipid metabolism through exosome (Exos)-mediated intercellular communication. Trem2-deficient macrophages exhibited enhanced Exos secretion, which were subsequently internalized by adjacent hepatocytes, functionally transferring regulatory miRNAs that reprogram lipid catabolic pathways (94).

#### 3.3.3 Renal metabolism

In the kidneys, the PKM2-driven aerobic glycolysis pathway serves as a critical regulator of macrophage activation and associated inflammatory responses. Functioning as a transcriptional coactivator, PKM2 forms a molecular complex with HIF-1α to upregulate glycolytic gene expression, which drives lactate overaccumulation while promoting HMGB1 acetylation-modulated extracellular release. And when the PKM-2 is inhibited by some gene, it can protect humans from dying in sepsis (95). And, it has been demonstrated that some patients with sepsis may develop anorexia. This anorexic state induces autophagy, a process that clears and recycles damaged cellular components while also regulating lipid metabolism, thereby enhancing cell survival (96). The activation of autophagy has been shown to exert a protective effect in the kidneys, during sepsis (97).

### 3.4 Barrier protection mechanisms

Maintaining the integrity of the endothelial barrier is an important organ protection mechanism during sepsis. Endothelial cells are pivotal in orchestrating the initial containment of infection (49). Multiple cell types and factors participate in this protective process through complex networks.

#### 3.4.1 Multicellular protection networks

Multicellular Collaborative Protection Mechanisms. B cells can modulate neutrophil function by regulating CXCR4, a mechanism that helps suppress neutrophil-mediated tissue damage. Although B cells can promote pro-inflammatory cytokines such as IL-6, protective models post-infection demonstrated reduced IL-6 concentrations in the circulation and liver, along with significantly decreased levels of key chemokines (CXCL1, CCL2) governing leukocyte trafficking in the peritoneum. This indicates the dual role of B cells in regulating inflammatory balance during sepsis (58).

#### 3.4.2 Lung barrier maintenance

In the lung, sepsis induces pathological alterations in alveolar epithelial and endothelial cells, thereby driving increased pulmonary permeability that culminates in edema formation (98). Dudakov et al. found that IL-22 can stimulate the proliferation of epithelial cells and suppress their apoptosis, facilitating tissue regeneration and repair after lung injury. This process begins with the activation of alveolar macrophages and CD11b+ dendritic cells via pattern recognition receptors (TLRs, NLRs, CLRs), secreting cytokines such as IL-1β, IL-23, IL-18, and IL-6. These cytokines subsequently activate NF-κB/STAT3 signaling in adaptive (CD4+ T cells) and innate lymphocytes (ILCs), driving RORγt-mediated transcriptional programming that effector cytokine production (IL-22). Thus, IL-22 exerts a dual function in pulmonary epithelial regeneration: promoting cell proliferation and enhancing tissue repair mechanisms post-injury (99).

#### 3.4.3 Renal barrier maintenance

In the kidneys, Chousterman et al. demonstrated that CX3CR1 deficiency results in diminished adhesion of inflammatory monocytes to the renal vascular endothelium, increased lesion formation, and elevated mortality rates, underscoring the protective role of CX3CR1 in renal sepsis pathophysiology (100). Endothelial cells represent one of the primary responders to sepsis-induced disturbances, rapidly undergoing molecular reprogramming to cope with pathological stressors. Equipped with intrinsic biomechanical sensing mechanisms, they swiftly detect and react to hemodynamic shifts—such as changes in vascular pressure and blood flow dynamics. In the renal microcirculation, sepsis disrupts perfusion patterns, resulting in impaired blood flow and occlusion of narrow capillaries by thrombi. Simultaneously, heightened vascular permeability drives fluid extravasation, leukocyte migration into tissues, and widespread molecular reorganization across tissue compartments. And these cells express a repertoire of receptors for cytokines, chemokines, DAMPs, and PAMPs, including Toll-like receptor 3 (TLR3) and TLR4. Engagement of these receptors initiates intracellular signaling cascades that drive phenotypic remodeling to counteract sepsis-induced dysfunction (101). The Angiopoietin-Tie2 signaling axis in vascular endothelium functions as a critical regulator of the transition from microvascular integrity to pathological disruption. Genetic ablation of Tie2 acutely compromises vascular barrier function *in vivo*, as demonstrated by experimental studies. In heterozygous Tie2 knockout models, partial deletion of Tie2 exacerbates vascular leakage and reduces survival rates after systemic endotoxin exposure or CLP procedures. These findings demonstrate that Tie2 exerts protective effects against sepsis-induced tissue injury (102).

#### 3.4.4 Gut barrier maintenance

In the gut, there is a kind of cell called ILCs. ILCs have been categorized as integral components of the innate immune system. These immune effectors are constitutively localized within mucosal interfaces, where they orchestrate early-phase immunological reactions, preserve epithelial barrier homeostasis, and drive developmental processes essential for lymphoid organ formation. And ILC3s affect releasing cytokines to protect guts against inflammation. Murine models with IL-22 deficiency develop exacerbated intestinal inflammatory responses accompanied by epithelial barrier dysfunction, culminating in accelerated mortality following bacterial challenge. Therefore, IL-22 could protect the tissue homeostasis in guts. Some findings demonstrate that lncKdm2b exerts indispensable regulatory control over the lineage stability of ILC3s (103). And scientists acclaimed that sepsis caused IL-33 secreted which stimulated ILC2s. With ILC2s, they could increase mucus production leading to an effect in protection. And it also depends on the IL-33–AREG–EGFR signaling pathway. With this mechanism, ILC2s exhibit dual cytoprotective functionality—modulating immune homeostasis through IL-13/IL-5-dependent anti-inflammatory effects while enhancing epithelial repair via amphiregulin-mediated proliferation—thereby preserving mucosal barrier integrity (104, 105).

Sepsis-induced multi-organ protection involves coordinated defense systems encompassing inflammatory regulation, immune homeostasis restoration, metabolic adaptation, and endothelial barrier maintenance. The body achieves dynamic balance between pro- and anti-inflammatory responses through cross-organ immune networks. Metabolically, organs maintain homeostasis via energy reprogramming and mitochondrial functional recovery, while endothelial integrity relies on spatiotemporal release of repair factors and inter-tissue cellular communication. Notably, protective mechanisms exhibit organ-specific and temporal characteristics—shifting from pathogen clearance in early phases to tissue repair in later stages. Although current studies have identified common regulatory frameworks, critical gaps remain in understanding inter-organ crosstalk, metabolic-epigenetic integration, and clinical translation. Future research should employ multi-omics integration and interdisciplinary approaches to develop precision therapies targeting disease tolerance mechanisms, ultimately advancing sepsis management paradigms.

## 4 Novel treatments and future direction

Conventional management strategies for sepsis, including time-sensitive antibiotic administration, volume resuscitation, and vasopressor-mediated hemodynamic stabilization, have been effective in early-stage sepsis resuscitation and achieve measurable improvements in patient prognosis through standardized resuscitation bundles (106). Nevertheless, these established therapeutic paradigms exhibit limited capacity to address the persistently elevated mortality rates observed in refractory septic shock cases. Consequently, the research focus has now shifted towards novel therapies based on the synergistic mechanisms of organ protection in sepsis, primarily encompassing inflammatory regulation, restoration of immune homeostasis, metabolic reprogramming, and endothelial barrier maintenance.

### 4.1 Mechanism-based therapies

#### 4.1.1 Therapies targeting inflammatory regulation

Systematic pathophysiological analysis confirms that organ protection can be achieved through the regulation of inflammatory responses. In the realm of inflammatory regulation, it is proved that using IL-10 is dramatically decreasing the key pro-inflammatory cytokines during sepsis. Adding apoptotic cell-stimulated macrophages (ASC-MΦ) also inhibited these cytokines. With rapamycin, it might enhance the autophagy to boost the protective role. And inspire by this mechanism, scientists created a broad-spectrum blood purification system which achieves continuous pathogen and toxin clearance in sepsis therapy through a biomimetic adsorption mechanism. In rat models of Staphylococcus aureus and Escherichia coli sepsis, the system significantly reduced bacterial burden in target organs, suppressed proinflammatory cytokine levels, and histopathological analysis confirmed attenuated multi-organ immune cell infiltration (107).

#### 4.1.2 Therapies aiming to restore immune homeostasis

Restoring immune homeostasis represents a pivotal protective strategy in sepsis management. Immune checkpoint modulation has emerged as a central therapeutic focus, particularly given the significant upregulation of immune checkpoint inhibitor proteins during septic responses, which directly contribute to the development of immunosuppressive states (108). Among these mechanisms, inhibition of the PD-1/PD-L1 axis has demonstrated notable clinical relevance. Preclinical evidence reveals that excessive PD-1 expression induces T cell exhaustion characterized by impaired proliferation and reduced secretion of critical cytokines (IL-2, IFN-γ, TNF-α) and chemokines. This immunosuppressive effect is most pronounced under low TCR stimulation conditions, while PD-1 activation also promotes T cell differentiation toward regulatory phenotypes (109). *In vivo* studies demonstrate that administration of anti-PD-1 or anti-PD-L1 antibodies significantly improves survival rates in wild-type (WT) bacterial sepsis models, with consistent efficacy observed across different infection types (110). Translational research further validates these findings: treatment with anti-PD-L1 antibodies in isolated immune cells from septic patients reduces T-cell apoptosis, enhances IFN-γ and IL-12 production, stimulates monocyte cytokine secretion, and improves neutrophil/NK cell functionality (111). Complementing these approaches, cytokine-based therapies like IFN-γ administration are being investigated to counteract immune paralysis. Notably, Dawulieti et al. demonstrated that cell-free DNA modulates Toll-like receptor 9 (TLR9) signaling pathways to reduce mortality in severe sepsis (112). In addition, the Cluster of differentiation 82 (CD82) functions as a co-stimulatory molecule in T cell activation and may also influence Treg activity. In septic mice, elevated CD82 expression is associated with Treg hyperactivation, potentially exacerbating sepsis-associated immunosuppression (113).

#### 4.1.3 Therapies focused on metabolic reprogramming

Cellular metabolism represents a third core mechanism for organ protection during sepsis. Melatonin is beneficial to adjust the function of mitochondrial cells by enhancing NADPH-cytochrome C reductase expression. Cannabinoids induce tolerogenic properties in human dendritic cells through metabolic pathways, by suppressing NF-κB activation, mitogen-activated protein kinase (MAPK) signaling, and mTOR activity while concurrently activating AMP-activated protein kinase (AMPK) and enhancing autophagy via cannabinoid receptor 1 (CB1) receptor- and PPARα-dependent mechanisms, they orchestrate metabolic reprogramming characterized by elevated mitochondrial oxidative phosphorylation (114).

#### 4.1.4 Therapies for endothelial barrier stabilization

Regarding direct endothelial barrier protection, studies have found that some agonists may have the effect, including Ang-1 and Tie-2 agonists, slit guidance ligand 2 N-terminal (Slit2N) agonists and sphingosine-1-phosphate receptor 1 (S1P1) agonists (115). These therapies aim to strengthen vascular endothelial cell junctions and reduce vascular leakage, thereby countering a core pathophysiological process in sepsis.

### 4.2 Diagnostics, precision medicine, and the role of artificial intelligence

Accurately defining sepsis is crucial for its rapid and accurate identification (116). As it has been discussed above, it is clinically difficult to diagnose sepsis early, which causes higher rates of mortality. Currently, some markers may promote the precision of diagnosing. Within innate immunity, subsets of natural killer (NK) cells hold remarkable diagnostic significance for sepsis. This is especially true when they are considered in combination with Bacteroides silverside and C-reactive protein (CRP). Moreover, within adaptive immunity, the phenotypes of T cells are closely associated with the severity of sepsis (83). Also, there are many markers have been proposed, such as Blimp1high dendritic cells, PDL1, PD-1^+^NK cells, B7-H1 expression on neutrophils, S100 proteins, HMGB1, Ang2/Tie2 system components which may reduce mortality of severe sepsis (35, 38, 117–120).

The integration of artificial intelligence (AI) and machine learning (ML) is revolutionizing sepsis management by bridging mechanistic research and clinical implementation. These tools are moving beyond retrospective analysis to become prospective, translational tools for prediction and early warning (121). The Targeted Real-time Early Warning System (TREWS) is a new machine learning model and the area under the curve (AUC) is 0.97, which can offer alerts to physicians and offer quicker treatment for patients with sepsis (122). The Epic Sepsis Model (ESM), a widely implemented penalized logistic regression model integrated into electronic health records (EHRs), is used in hundreds of U.S. hospitals to automatically flag patients at risk of sepsis (123, 124). A Smart Sepsis Predictor (SSP) model based on a Recurrent Neural Network (RNN) architecture has demonstrated high predictive accuracy within a critical 12-hour window by identifying complex patterns in vital signs and laboratory data, enabling timely clinical alerts (125). Given the complexity of the sepsis response, panels of biomarkers or models that integrate biomarkers with clinical data are necessary, alongside specific data analysis methods—most of which fall under the scope of machine learning. Evaluation using non-routine datasets to characterize gene expression, inflammatory and metabolomic sepsis phenotypes has further refined these analyses, providing critical insights into the use of therapies such as steroids. The research highlighted herein suggests several potential future applications: these tools, when combined with routine clinical and biological information, could enhance the timeliness and accuracy of diagnosis, reveal relevant physiological pathways and therapeutic targets, facilitate targeted recruitment for clinical trials, and optimize clinical management (126). And the machine learning model also delivered reliable performance in predicting sepsis across 12-, 24-, and 48-hour time intervals, achieving high AUROC while maintaining low false positive and false negative rates. These findings are clinically significant, as they confirm the model’s consistent predictive capability across critical early-warning windows (127).

Beyond prediction, AI is pivotal in advancing precision medicine by deciphering sepsis heterogeneity. The integration of multi-omics advancements and point-of-care (POC) diagnostics with mechanistic insights into sepsis pathophysiology heralds the emergence of precision immunotherapy tailored to individual immunophenotypic profiles (36).

AI also extends to optimizing treatment decisions and predicting outcomes. There is a new artificial intelligence clinician. When clinicians’ actual treatment deviated from the AI Clinician’s recommended policy, it most commonly involved the administration of insufficient vasopressor. Early application of low-dose vasopressor has been proposed as beneficial in sepsis management, as it may prevent the administration of excessive fluid volumes—a factor associated with poorer outcomes. And the findings support this approach, while importantly enabling the personalization of treatment for each patient. The integration of this AI clinician could significantly reduce sepsis mortality (128). Even a modest decline in sepsis-related death rates would translate to the preservation of thousands of lives worldwide annually (129).

### 4.3 Current challenges and future prospects

Substantial knowledge gaps persist in sepsis therapeutics, necessitating focused scientific inquiry to optimize clinical efficacy. The septic host response manifests as a dynamic interplay of exquisitely regulated nonlinear dynamics, which is hard to be captured by the single timepoints (130). As the sepsis mouse model, the CLP model was utilized given its straightforward methodology and capacity to replicate polymicrobial infection dynamics, establishing its recognition as the most clinically relevant experimental surrogate for human sepsis and the gold standard. Nevertheless, this model presents inherent operational constraints, particularly technical challenges in standardizing caecal content dispersion (24). Although inflammation during sepsis is the main mechanism to overcome bacterial infection, anti-inflammatory tolerance mechanisms may inadvertently promote autoimmune disorders and carcinogenesis. Consequently, clinical investigations require rigorous risk-benefit evaluation on a case-specific basis (79). Additionally, emerging evidence supports distinct signaling cascades underlying Gram-negative versus Gram-positive induced septic shock, necessitating pathogen-class-specific therapeutic strategies aligned with their pathophysiological divergence (131). Sepsis survivors frequently develop persistent physical morbidity and neurocognitive deficits, with demonstrating functional decline extending beyond hospital discharge. Furthermore, analyses reveal elevated risks persist for secondary infections, major adverse cardiovascular events, acute renal failure, and aspiration pneumonia. These findings underscore the critical need for multidisciplinary surveillance strategies addressing the post-sepsis syndrome continuum (132). As for the organ protection, current experimental comprehension of these pathways remains nascent, with clinical translatability yet to be established. And different organs have their own tolerance capacities which is difficult to determine the standard of tissue damage (133). We still have limitations of the concept of sepsis, although it has been upgraded with the time. And there is the heterogeneity ranging from sepsis, patients to the treating team. All these things cause it difficult to do the clinical trials (134). Immunotherapeutics exhibit robust preclinical therapeutic potential, while their clinical translation remains constrained by insufficient mechanistic elucidation; the absence of validated biomarkers for guiding personalized therapeutic regimens further compounds these translational challenges (135).

Future directions demand multidisciplinary collaboration to elucidate underlying mechanisms and develop precision frameworks that integrate acute care with long-term recovery, shifting from “one-size-fits-all” approaches to dynamic, immunophenotype-guided management strategies.

## 5 Conclusion

This review has comprehensively discussed the mechanisms of multi-organ dysfunction caused by sepsis and the various protection mechanisms that different organs employ to counteract sepsis-induced damage. The information presented is based on previous research on sepsis. Additionally, novel treatment perspectives have been introduced, providing a roadmap for future research on sepsis management.

Despite some positive results from sepsis awareness campaigns, the incidence of sepsis continues to rise. This highlights the need for further education and knowledge dissemination. Sepsis should be regarded as a medical emergency. The continuous shortening of the timeframes in sepsis care bundles underscores the critical role of emergency physicians in identifying and initiating emergency resuscitation and treatment for septic patients. Ongoing education, clinical research, and strict adherence to recommendations and guidelines are essential for effectively treating sepsis and reducing its mortality rate.

## Glossary

ADRB2: β2-adrenergic receptor
AhR: Aryl hydrocarbon receptor
AI: Artificial intelligence
ALI: Acute lung injury
AMP: Adenosine monophosphate
AMPK: AMP-activated protein kinase
Ang: Angiopoietin
APC: Activated protein C
ASC-MΦ: Adipose-derived mesenchymal stromal cells conditioned media
ATP: Adenosine triphosphate
CB1: Cannabinoid receptor 1
CD39: Cluster of differentiation 39
CD62E: Monocyte-associated E-selectin
CD82: Cluster of differentiation 82
CLP: Cecal ligation and puncture
CNS: central nervous system
CRP: C-reactive protein
DAMPs: Danger-associated mo
EC-EVs: Extracellular vesicles
eGC: Endothelial glycocalyx
EHRs: Electronic health records
ENO1: Enolase 1
ERK: Extracellular signal-regulated kinases
ESM: Epic Sepsis Model
Exos: Exosome
FA: Fatty acid
FGF9: Fibroblast growth factor 9
FTH: Ferritin H chain
G6Pase: Glucose-6-phosphatase
HIF-1α: Hypoxia-inducible factor 1-alpha
HLA-DR: Human leukocyte antigen D-related
HMGB1: High-mobility group B1
IFITM2: Interferon-induced transmembrane protein 2
ILCs: Innate lymphocytes
IFN-γ: Interferon-gamma
LPS: Lipopolysaccharide
MAPK: Mitogen-activated protein kinase
Mcp-1: Monocyte chemoattractant protein-1
MDSCs: Myeloid-derived suppressor cells
ML: Machine learning
MPO: Myeloperoxidase
MSCs: Mesenchymal stem cells
mTOR: mammalian target of rapamycin
NADPH: Nicotinamide adenine dinucleotide phosphate
NE: Norephinephrine
NETs: Neutrophil extracellular traps
NK: Natural killer
NKT: Natural killer T
PAMPs: Pathogen-associated molecular patterns
PD-1: Programmed cell death receptor-1
PD-L1: Programmed death-ligand 1
POC: Point-of-care
PPARα: Peroxisome proliferator-activated receptor alpha
PRRs: Pattern recognition receptors
RAP1B: RAS-associated protein 1B
RNN: Recurrent Neural Network
ROS: Reactive oxygen species
S1P1: Sphingosine-1-phosphate receptor 1
SI-ALI: Sepsis-induced acute lung injury
SIRS: Systemic Inflammatory Response Syndrome
Slit2N: Slit guidance ligand 2 N-terminal
SSP: Smart Sepsis Predictor
TECs: Tubular epithelial cells
TF: Tissue factor
Tie-2: Tyrosine kinase with immunoglobulin-like and EGF-like domains 2
TLR4: Toll-like receptor-4
TLR9: Toll-like receptor 9
TLRs: Toll-like receptors
TREWS: Targeted Real-time Early Warning System
Tregs: Regulatory T cells
TTP: Tristetraprolin
VWF: Von willebrand factor
WT: Wild-type.
